# Spectrophotometric and Reversed-Phase High-Performance Liquid Chromatographic Method for the Determination of Doxophylline in Pharmaceutical Formulations

**DOI:** 10.4103/0975-1483.66791

**Published:** 2010

**Authors:** HR Joshi, AH Patel, AD Captain

**Affiliations:** *Shree Swaminarayan College of Pharmacy, Kalol Highway Road, Kalol - 382 721, Gujarat, India*; 1 *Torrent Research Centre, Bhat, Dist.: Gandhinagar - 382 428, Gujarat, India*; 2 *A.R. College of Pharmacy and Institute of Science and Technology for Advance Research (I.S.T.A.R), Vallabh Vidyanagar - 388 120, Gujarat, India*

**Keywords:** Doxophylline, HPLC, reversed-phase, UV-spectrophotometry

## Abstract

Two methods are described for determination of Doxophylline in a solid dosage form. The first method was based on ultraviolet (UV)-spectrophotometric determination of the drug. It involves absorbance measurement at 274 nm (λ_max_ of Doxophylline) in 0.1 N hydrochloric acid. The calibration curve was linear, with the correlation coefficient between 0.99 and 1.0 over a concentration range of 0.20–30 mg/ml for the drug. The second method was based on high-performance liquid chromatography (HPLC) separation of the drug in reverse-phase mode using the Hypersil ODS C_18_ column (250 × 4.6 mm, 5 mm). The mobile phase constituted of buffer acetonitrile (80:20) and pH adjusted to 3.0, with dilute orthophosphoric acid delivered at a flow rate 1.0 ml/min. Detection was performed at 210 nm. Separation was completed within 7 min. The calibration curve was linear, with the correlation coefficient between 0.99 and 1.0 over a concentration range of 0.165–30 mg/ml for the drug. The relative standard deviation was found to be <2.0% for the UV-spectrophotometry and HPLC methods. Both these methods have been successively applied to the solid dosage pharmaceutical formulation, and were fully validated according to ICH guidelines.

## INTRODUCTION

**Figure 1:**
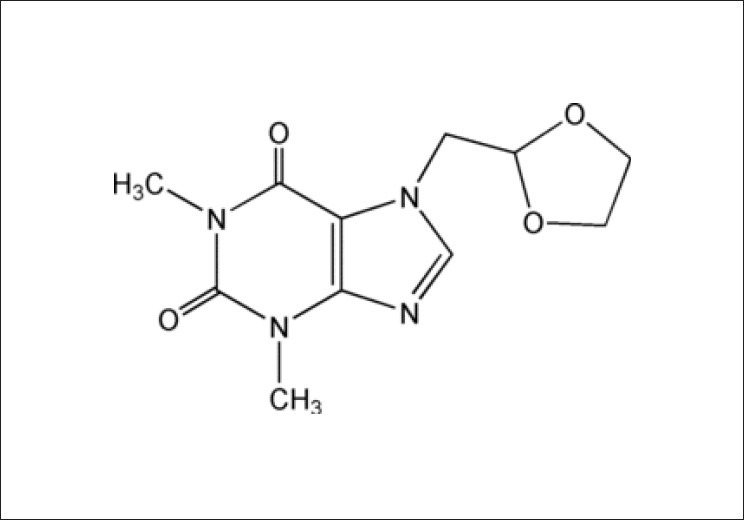
Structure of Doxophylline

Doxophylline is chemically designated as 7(1, 3 dioxolone-2-yl methyl) theophylline. Presence of a dioxolane group in position 7 differentiates it from theophylline.[1] The chemical structure of Doxophylline is provided herewith [Figure 1].[2]

It is a new antibronchospastic drug recently introduced in therapy, with pharmacological properties like theophylline, a potent adenosine receptor antagonist. Doxophylline does not affect gastric acid secretion, either *in vivo* or *in vitro*, unlike theophylline. The lack of side-effects with doxophylline indicates that the drug can be used safely and effectively in the treatment of chronic obstructive pulmonary disease (COPD).[3] Doxophylline inhibits phosphodiesterase (PDE IV) activities with the consequent increase of cyclic AMP, which determines relaxation of the smooth musculature. Doxophylline appears to have decreased affinities toward adenosine A1 and A2 receptors, which may account for the better safety profile of the drug. Doxophylline does not interfere with calcium influx into the cells or antagonize calcium channel blockers.[4] Unlike aminophylline, it has low secretagogue activity and is suitable for asthmatic patients with peptic ulcer disease.[5]

Doxophylline is indicated for the treatment of bronchial asthma and COPD.[6]

Some analytical methods for quantitative determination of Doxophylline in pharmaceutical formulations are described in the literature, like ultraviolet (UV)-spectrophotometry[7] and LC-MS (Liquid Chromatography-Mass Spectroscopy).[8–10] At present, no high-performance liquid chromatography (HPLC) and UV-spectrophotometric methods are reported for the estimation of Doxophylline in a tablet dosage form. The purpose of this work is to develop and validate the proposed methods for routine analysis in a quality control laboratory.

## EXPERIMENTAL PROCEDURE

### Instrument and condition

1. UV-visible spectrophotometer - Model UV-1700 (Shimadzu, Tokyo, Japan).
2. HPLC system - Shimadzu LC 2010C integrated system equipped with quaternary gradient pump, 2010C UV-VIS detector, 2010C column oven and 2010C programmable auto sampler controlled by CLASS-VP software. (SHIMADZU USA Manufacturing Inc, 1900, SE 4^th^ Ave, Canby, OR, 97013-4348, North America, USA)
3. Analytical column - Hypersil ODS C_18_ (250 × 4.6 mm, 5 mm particle size), (Weber Consulting, Attila u. 38/b. H-2132 Göd, Hungary)
4. Detector - UV visible
5. Chromatographic parameters- Detection at 210 nm, flow rate 1.0 ml/min.
6. Mobile phase - Potassium dihydrogen phosphate (pH 3.0 ± 0.2 adjusted with orthophosporic acid)–acetonitrile (80:20, v/v).
7. Diluent - 0.1 N hydrochloric acid.

### Reagents

1. Doxophylline reference standard - Assigned purity 99.24% (Cadila Healthcare Limited, Ankleshwar, Gujarat, India).
2. Acetonitrile - AR grade (Spectrochem), Spectrochem Private Limited, Office 221, 2nd Floor, Anand Bhuvan, 17, Babu Genu Road, Princess Street, MUMBAI - 400 002.
3. Orthophosphoric acid - AR grade (E-Merck Limited), E-Merck (India) Ltd, Shiv Sagar Estate, ‘A’, Dr. A B Road, Worli, Mumbai, 400018, India
4. Commercially available Doxophylline tablet - Claimed to contain 800 mg of the drug. Procured from Zydus Cadila, Ahmedabad, Gujarat, India.

### Standard preparation

#### For UV-spectrophotometric and HPLC methods

Standard stock solution of 400 μg/ml was prepared by dissolving 40 mg working standard of Doxophylline in 100 ml of diluent. The working standard solution of Doxophylline had a final concentration of 20 μg/ml and was prepared by appropriate dilution from the stock solution.

### Sample preparation

#### For UV-spectrophotometric and HPLC methods

Twenty tablets were weighed and crushed into fine powder. An accurately weighed quantity of powder equivalent to about 125 mg of Doxophylline was transferred into a 250 ml volumetric flask. Add 100 ml of diluent and sonicate it for 30 min with continuous shaking. Make the volume up to the mark with 0.1 N HCl. This solution was filtered through a 0.45 μm HVLP nylon filter. Make an appropriate dilution to get the final concentration of Doxophylline 20 μg/ml. Appropriated aliquots were subjected to the above methods and the amount of Doxophylline was determined.

### UV-spectrophotometric method

#### Construction of the calibration curve

λ_max_ of Doxophylline (20 μg/ml) was determined by scanning the drug solution in diluent and was found to be at 274 nm. To construct Beer’s plot for Doxophylline, dilutions were made in diluent using stock solution at different concentration (4, 12, 16, 20, 24, and 30 μg/ml) levels. The drug followed linearity within the concentration range of 4–30 μg/ml.

#### Assay of the tablet formulation

Twenty tablets were weighed and crushed into fine powder. An accurately weighed quantity of powder equivalent to about 125 mg of Doxophylline was transferred into a 250 ml volumetric flask. Add 100 ml of diluent and sonicate it for 30 min with continuous shaking. Make the volume up to the mark with 0.1 N HCl. This solution is then filtered through a 0.45-μm HVLP (High Vinyl Liquid Polymer) nylon filter. Make appropriate dilution to get the final concentration of Doxophylline 20 μg/ml. Appropriated aliquots were subjected to the above methods and the amount of Doxophylline was determined.

### HPLC Method

#### Construction of the calibration curve

To construct Beer’s plot for Doxophylline, dilutions were made in the diluent using stock solutions at different concentration (4, 12, 16, 20, 24 and 30 μg/ml)levels. The drug followed linearity within the concentration range of 4–30 μg/mlfor Doxophylline at 210 nm.

#### Assay of the tablet formulation

Twenty tablets were weighed and crushed into fine powder. An accurately weighed quantity of powder equivalent to about 125 mg of Doxophylline was transferred into a 250-ml volumetric flask. Add 100 ml of diluent and sonicate it for 30 min with continuous shaking. Make the volume up to the mark with 0.1 N HCl. This solution was filtered through a 0.45-μm HVLP nylon filter. Make appropriate dilution to get the final concentration of Doxophylline 20 μg-ml. Appropriated aliquots were subjected to the above methods and the amount of Doxophylline was determined.

## RESULT AND DISCUSSION

### System suitability and system precison (For HPLC)

This parameter has been performed before starting any validation parameter each time. The purpose of this parameter is to ensure that system is working properly and it can be used further for analysis and validation. For more details, Table 1.

**Table 1:** System suitability and system precision (for HPLC)

Compound	Retention time (Mean ± SEM)	*n*	T	k’
Doxophylline	6.434 ± 0.06217	11034.808	1.22	642.4

*n* = Theoretical plates

T = Asymmetry

k’ = Capacity factor

### Linearity

**Figure 2:**
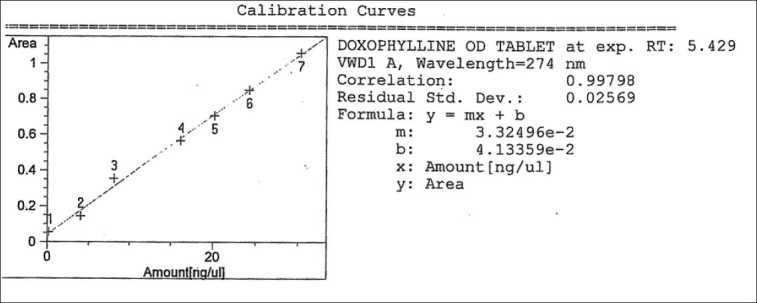
Calibration curve for Doxophylline (for the UV-spectrophotometric method)

**Figure 3:**
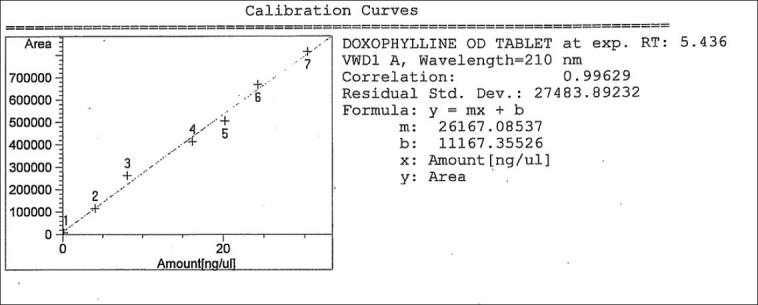
Calibration curve for Doxophylline (high-performance liquid chromatography)

The plot of absorbances against concentration is shown in Figures 2 and 3. It can be seen that the plot is linear over the concentration range of 0.20–30 μg-ml in UV-spectrophotometry and 0.165–30 μg/ml in HPLC for Doxophylline, with correlation coefficients (r^2^) of 0.99798 and 0.99629, respectively. The obtained results are presented in Tables 2A and 2B.

**Table 2A:** Characteristics of the Analytical method derived from the standard calibration curve (for UV-spectrophotometric method)

Compound	LOD μg/ml	LOQ μg/ml *n* = 5	Linearity range μg/ml	Correlation coefficient r^2^	Residual standard regression σ	Slope of regression S
Doxophylline	0.07	0.2	4–30	0.99798	0.02569	0.00332

LOD = Limit of detection

LOQ = Limit of quantification

**Table 2B:** Characteristics of the analytical method derived from the standard calibration curve (for HPLC method)

Compound	LOD μg/ml	LOQ μg/ml n = 5	Linearity range μg/ml	Correlation coefficient r^2^	Residual standard regression σ	Slope of regression S
Doxophylline	0.05	0.165	4–30	0.99629	27483.89232	26167.08537

LOD = Limit of detection

LOQ = Limit of quantification

### Standard and sample solution stability

**Figure 4:**
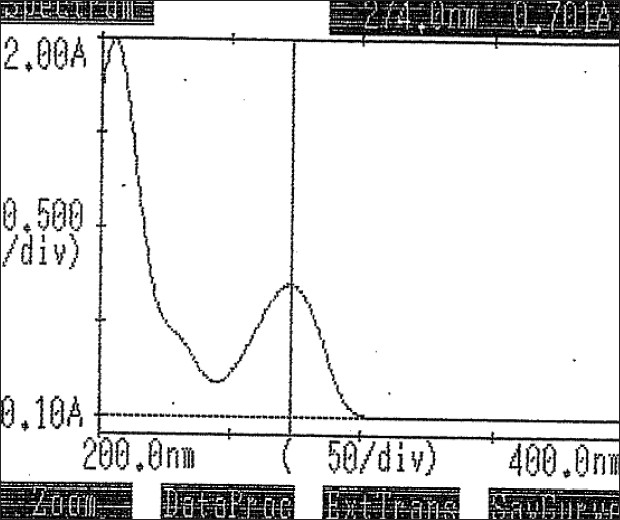
Spectrum of Doxophylline (20 μg/ml) in 0.1 N hydrochloric acid by the ultravioled-visible spectrophotometer

**Figure 5:**
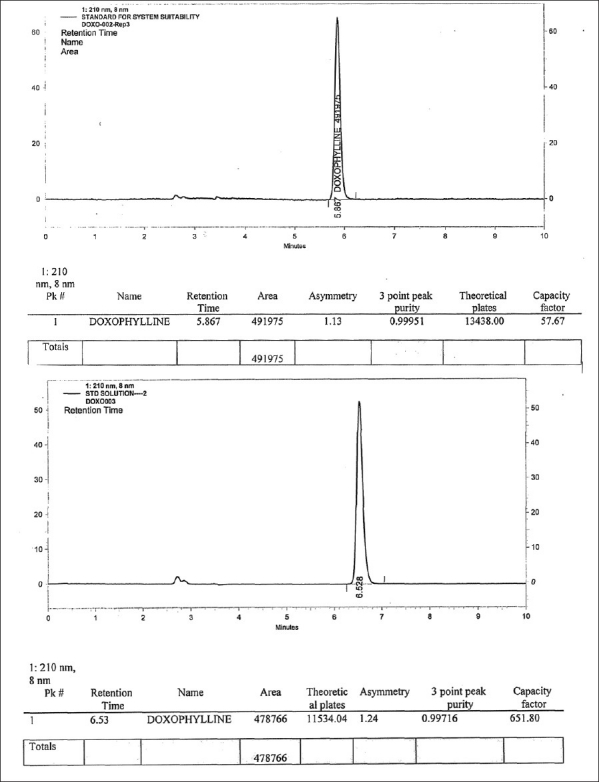
Chromatogram of the standard solution

**Figure 6:**
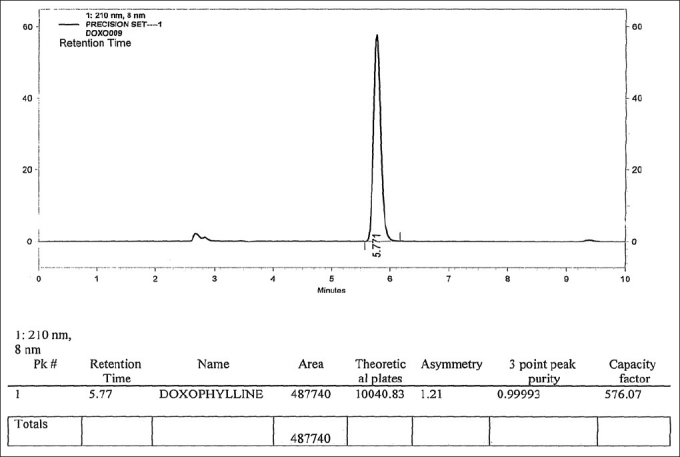
Chromatogram of the sample solution

Standard and sample solution stabilities were evaluated at room temperature for 48 h. The relative standard deviation (RSD) was found to be below 2.0%. It shows that the standard and sample solutions were stable up to 48 h at room temperature. Please refer, Spectrum of Standard which is provided as Figure 4 and Chromatograms of Standard and sample which are provided as Figures 5 and 6 respectively.

### Method precision

The RSD for six replicates of the sample solution was <2.0%, which met the acceptance criteria established for the spectrophotometric and HPLC methods. The obtained results are presented in Tables 3A and 3B.

**Table 3A:** Method precision (for UV-spectrophotometric method)

Compound	Concentration μg/ml (n = 6)	Absorbance Mean ± SEM (n = 6)	% assay Mean ± SEM (n = 6)	% RSD (n = 6)
Doxophylline	20	0.716 ± 0.00204	101.75 ± 0.3535	0.9

**Table 3B:** Method precision (for HPLC method)

Compound	Concentration μg/ml (n = 6)	Retention time Mean ± SEM (n = 6)	% assay Mean ± SEM (n = 6)	% RSD (n = 6)
Doxophylline	20	5.62 ± 0.0460	101.0 ± 0.4232	1.0

### Accuracy

Accuracy was performed at three levels: 50, 100 and 150%. Percentage recovery and low RSD value show the accuracy of the spectrophotometric and HPLC methods. The data are presented in Tables 4A and 4B.

**Table 4A:** Method accuracy (for UV–spectrophotometric method)

Level	Drug added (mg)	Drug recovered (mg)	% assay (Mean ± SEM) (*n* = 3)	% RSD of assay (*n* = 3)
Doxophylline				
50%	62.05	62.41	100.6 ± 0.088	0.2
100%	124.01	123.75	99.8 ± 0.409	0.7
150%	185.88	186.23	100.3 ± 0.266	0.5

**Table 4B:** Method accuracy (for HPLC method)

Level	Drug added (mg)	Drug recovered (mg)	%assay (Mean ± SEM) (n = 3)	% RSD of assay (n = 3)
Doxophylline				
50%	62.05	62.21	100.26 ± 0.448	0.8
100%	124.01	123.41	99.5 ± 0.458	0.8
150%	185.88	186.27	100.2 ± 0.321	0.6

### Method ruggedness

Ruggedness test was determined between two different analysts, instruments and columns. The value of RSD below 2.0% showed ruggedness of the developed spectrophotometric and HPLC methods. The results of ruggedness are presented in Tables 5A and 5B.

**Table 5A:** Method ruggedness (for UV-spectrophotometric method)

Compound	% assay Mean ± SEM (n = 6)	% RSD of assay (n = 6)
Day 1, Analyst-1, Instrument-1 Doxophylline	101.75 ± 0.3535	0.9
Day 2, Analyst-2, Instrument-2 Doxophylline	101.01 ± 0.1973	0.5

**Table 5B:** Method ruggedness (for HPLC method)

Compound	% assay Mean ± SEM (n = 6)	% RSD of assay (n = 6)
Day 1, Analyst-1, Instrument-1, Column-1 Doxophylline	101.0 ± 0.4232	1.0
Day 2, Analyst-2, Instrument-2, Column-2 Doxophylline	100.05 ± 0.20125	0.5

### Method robustness

The method was found to be robust as small but deliberate changes in the method parameters had no detrimental effect on the method performance, as shown in Table 6. The content of the drug was not adversely affected by these changes, as evident from the low value of RSD, indicating that the method is robust.

**Table 6:** Method robustness (for HPLC method

Compound	% RSD in normal	Changed condition (n = 5)	
Temperature	% RSD normal	% RSD (-5°C)	% RSD (+5°C)
Doxophylline	1.0	0.18	1.05
pH	% RSD normal	% RSD (-0.2 unit)	% RSD (+0.2 unit)
Doxophylline	1.0	0.19	0.55
Flow rate	% RSD normal	% RSD (-10%)	% RSD (+10%)
Doxophylline	1.0	0.10	0.19
Mobile phase ratio	% RSD normal	% RSD (-2%)	% RSD (+2%)
Doxophylline	1.0	0.08	0.19

### Specificity

There was no interference from sample placebo, and peak purity of Doxophylline was 0.99629. This indicates that the developed analytical method was specific for its intended purpose.

## DISCUSSION

### For UV-spectrophotometric method

The proposed analytical method is simple, accurate and reproducible. Doxophylline showed λ_max_ at 274 nm. The advantages lie in the simplicity of sample preparation and the cost economic reagents used. The contribution of another important factor is its limit of detection (LOD). Results from statistical analysis of the experimental results were indicative of satisfactory precision and reproducibility. Hence, this spectrophotometric method can be used for analysis of different solid dosage formulations in commercial quality control laboratories.

### For HPLC

Considering the efficiency of HPLC, an attempt has been made to develop simple, accurate, precise, rapid and economic methods for estimation of Doxophylline in a solid dosage form. Thus, the method described enables quantification of Doxophylline. The advantages lie in the simplicity of sample preparation and the cost-economic reagents used. The contribution of another important factor is its LOD. Results from statistical analysis of the experimental results were indicative of satisfactory precision and reproducibility. Hence, this HPLC method can be used for the analysis of different solid dosage formulations in commercial quality control laboratories.

The comparative advantages and disadvantages of the UV-spectrophotometric method and reverse-phase HPLC method has been provided herewith.

**Table 7:** Comparison between UV and HPLC Method

Parameter	UV method	HPLC method
Mechanism	Measurement of absorbance of samples containing only one absorbing component	Measurement of absorbance and separation (partition) of samples containing more than one absorbing component at a time
Accuracy and precision	Low compared to the RP-HPLC method	Very accurate and precise
Cost of analysis	Very low	High
Reagents/solvents/diluents/mobile phase	Use of a polar solvent generally is sufficient	Use of mobile phase having a combination of either buffer and polar solvent and/or use of two polar solvents
Analysis of compounds	Polar substances having λ_max_ between 200 and 400 nm	Substances can be analyzed beyond the limit provided in the UV method due to the wider variety of the detector being employed
Sensitivity	Limited in sensitivity	Greater sensitivity (as various detectors can be employed)
Instrumentation	Easy to operate	Compared to the UV method, complex to operate
Speed	Compared to HPLC, analysis can be completed within lesser time	Time required for analysis depends on the nature of the molecule to be analyzed
Resolution	Low resolution compared to the HPLC method. Required to go for first and second derivative spectrophotometric methods	Greater resolution (wide variety of stationary phases)
Type of test/analysis	It can be use as a confirmatory test for a particular compound	It is used as a specific identification test for a particular compound
Useful at scale	Useful at laboratory scale at the primary level	Useful at a large scale, where complex molecules have to be analyzed
Applications	Useful to find out the qualitative parameter, like λ_max_ of a particular compound	Useful to find out the quantitative parameters, like retention time of a particular compound
Degradation/by products	Can be analyzed simultaneously	Can be analyzed within one analysis
Calculation	Calculations have to be performed manually based on the λ_max_ of a particular compound	Calculations are performed by the integrator itself
